# Mechanism of N6-methyladenosine (m6A)-mediated upregulation of LINC00958 in the growth of cervical cancer

**DOI:** 10.3389/fonc.2026.1663904

**Published:** 2026-03-06

**Authors:** Yuting Li, Zhengying Liu, Hongling Jin

**Affiliations:** Department of Gynecology, Southern Central Hospital of Yunnan Province, Honghe, Yunnan, China

**Keywords:** BAG3, cervical cancer, c-MYC, LINC00958, m^6^A modification, METTL3

## Abstract

**Objective:**

Cervical cancer (CC) remains a prominent contributor to cancer mortality amongst women. Long non-coding RNAs (LncRNAs) participate in CC progression. This study probed into the potential mechanism of LINC00958 in CC growth.

**Materials and methods:**

LINC00958 expression in CC tissues and cells was determined. The correlation between LINC00958 expression and CC prognosis was analyzed. LINC00958 expression was interfered in CC cells, followed by assessment of CC cell proliferation and apoptosis. An xenograft tumor model was established in nude mice. METTL3, c-MYC, and BAG3 expression was determined. m6A level was quantitatively analyzed, and the level of m6A-modified LINC00958 was detected. The binding of LINC00958 to c-MYC, as well as the binding of c-MYC to BAG3 were confirmed. Functional rescue experiments were designed to verify the effect of METTL3/BAG3 on CC growth.

**Results:**

LINC00958 expression was elevated in CC and correlated with the prognosis and clinicopathological features of CC patients. LINC00958 silencing suppressed CC cell proliferation and facilitated apoptosis *in vitro*, and repressed tumor growth *in vivo*. Mechanically, METTL3-mediated m6A modification elevated LINC00958 expression by promoting LINC00958 stability. LINC00958 activated the transcriptional activity of c-MYC, and c-MYC bound to BAG3. METTL3 overexpression or BAG3 overexpression offset the impact of LINC00958 silencing on CC growth.

**Conclusion:**

METTL3-mediated m6A modification elevated LINC00958 expression. LINC00958 enhanced CC cell proliferation but depressed apoptosis via the c-MYC/BAG3 axis.

## 1 Introduction

Cervical cancer (CC) constitutes a prominent cause of cancer-related mortality in women throughout the world (1). It is well established that CC is closely associated with human papillomavirus infection, while additional genetic and epigenetic alternations are also required for CC progression (2). Currently, the available therapeutic strategies for CC include surgery (pelvic lymphadenectomy and radical hysterectomy), chemotherapy, and radiotherapy (3). Unfortunately, the clinical outcomes are still far from expectation because a considerable number of CC patients are not diagnosed until they have progressed to the advanced stage (4). In recent years, the incidence of CC is still on the rise in developing countries due to the lack of potent prevention and screening methods (5). Therefore, it is imperative to explore novel therapeutic targets and prognostic biomarkers to improve the outcomes of CC patients.

Recent researches focus on the aberrant alternations of long non-coding RNAs (lncRNAs) in diverse human malignancies (2). LncRNAs are a class of transcripts with over 200 nucleotides, which are emerging as crucial mediators of tumorigenesis and progression (6). The current knowledge has highlighted the role of dysregulated lncRNAs in CC in terms of tumorigenesis, invasion, metastasis, treatment resistance, and prognosis (7). Long non-coding RNA 00958 (LINC00958) is initially recognized as an oncogene of bladder cancer (8). Subsequently, numerous studies have depicted that LINC00958 also exerts notable effects on the biological processes of many other cancers such as glioma (9) and gastric cancer (10). Importantly, LINC00958 shows a dramatically surge in CC (11). However, the knowledge regarding the underlying mechanism of LINC00958 in CC growth is unclear.

N^6^-methyladenosine (m^6^A), as the most abundant internal modification in lncRNAs, participates in the modulation of RNA stability and mRNA translation efficiency (12, 13). Since m^6^A modification profoundly affects multiple biological processes, abnormal m^6^A modification contributes to tumorigenesis (14). Commonly, m^6^A is catalyzed by the methyltransferase, represented by methyltransferase-like 3 (METTL3) (13). METTL3 has been extensively reported as an oncogene to trigger the initiation and progression of various cancers by depositing m6A modification on critical transcripts (15, 16). For example, m^6^A modification initiated by METTL3 can facilitate YAP translation to enhance metastasis and drug resistance in lung cancer (17). METTL3-mediated m^6^A modification participates in epithelial-mesenchymal transition (EMT) in gastric cancer (18). Notably, METTL3 is also demonstrated to modulate m^6^A modification in LINC00958, thereby increasing its RNA stability and elevating LINC00958 expression in hepatocellular carcinoma (19). Accordingly, we hypothesize that METTL3-mediated m^6^A modification can affect LINC00958 expression in CC. Hence, this study aims to investigate the specific mechanism of LINC00958 in CC growth. Our findings reveal for the first time that METTL3-mediated m6A enhances LINC00958 expression, and then LINC00958 drives CC growth via the c-MYC/BAG3 axis. This provides mechanistic insights and potential therapeutic targets with significant conceptual relevance for CC treatment.

## 2 Materials and methods

### 2.1 Ethics statement

This study was performed following the approval of the Ethical Committee of Southern Central Hospital of Yunnan Province and complied with the *Declaration of Helsinki*. All patients had signed the informed consent. Animal experiments were implemented based on the *Guide for the Care and Use of Laboratory Animals* (20).

### 2.2 Clinical tissue collection

The CC tissues and paracancerous tissues were collected from 70 CC patients (aged 38–65 years, at an average age of 51.40 years) who underwent surgical resection in Southern Central Hospital of Yunnan Province. These patients did not receive any radiotherapy or chemotherapy before the operation, with complete clinical data. The tissues were frozen in liquid nitrogen immediately after the operation and kept at -80°C for subsequent experimentation. These patients were followed up by means of telephone or return visit. The detailed clinicopathological features and 5-year overall survival rate were recorded.

### 2.3 Cell culture

CC cells (SiHa, HeLa, C-33A, MS751, and CaSki) and human normal cervical epithelial cells (Ect1/E6E7) (ATCC, Manassas, Virginia, USA) were cultured in RPMI-1640 medium (GIBCO, Grand Island, NY, USA) containing 10% fetal bovine serum (Beyotime, Beijing, China). When the cells attained 70-80% confluence, transfection experiments were conducted.

### 2.4 Cell treatment

The lentiviral overexpression vectors of METTL3 (LV-oe-METTL3) and negative control (LV-oe-NC) were obtained from Genechem (Shanghai, China). HeLa cells were infected with 5 mg/mL polybrene and lentivirus for 48 h. The titer of lentivirus (3 × 10^8^ PFU/mL) was determined using the fluorescent activated cell sorting method. The stably transfected cells were screened using 5 μg/mL puromycin (Sigma-Aldrich, Merck KGaA, Darmstadt, Germany). Three short hairpin RNA (shRNA) sequences for LINC00958 (sh-LINC00958#1, sh-LINC00958#2, and sh-LINC00958#3), pcDNA3.1 LINC00958 (pc-LINC00958), pcDNA3.1 B-cell lymphoma 2 (Bcl-2)-associated athanogene 3 (BAG3) (pc-BAG3), three shRNA sequences for METTL3 (sh-METTL3#1, sh-METTL3#2, and sh-METTL3#3), three shRNA sequences for c-MYC (sh-c-MYC#1, sh-c-MYC#2, and sh-c-MYC#3), and their NCs obtained from GenePharma (Shanghai, China) were transfected into SiHa or HeLa cells using Lipofectamine 2000 (Invitrogen, Carlsbad, CA, USA). The subsequent experiments were conducted after 48 h.

### 2.5 Cell counting kit-8 assay

The treated SiHa or HeLa cells were rinsed with phosphate-buffered saline (PBS). Then, cell proliferation was measured using the CCK-8 kit (Dojindo Laboratories, Kumamoto, Japan). The cells were seeded into 96-well plates (2000 cells/well) and cultured at 37°C with 5% CO_2_ for 0, 24, 48, and 72 h. Subsequently, 10 μL CCK-8 solution was added for another 3 h incubation. The absorbance was examined at 450 nm using a microplate reader (Bio-Rad, Hercules, CA, USA).

### 2.6 Colony formation assay

The treated SiHa or HeLa cells were collected, resuspended in RPMI-1640 medium, and counted. Then, cells were seeded into 6-well plates (150 cells/well) and cultured for 14 d. Afterward, the medium was sucked off and cells were fixed with 4% paraformaldehyde (Beyotime) for 20 min. Finally, the cells were stained with 200 μL crystal violet solution (Beyotime) for 15 min. The number of staining colonies was observed and counted under an inverted microscope (Nikon, Tokyo, Japan).

### 2.7 Flow cytometry

The treated SiHa or HeLa cells were seeded into a 6-well plate (1 × 10^6^ cells/well) and cultured at 37°C with 5% CO_2_ (21). After 24 h of incubation, the cells were harvested using trypsin and washed with pre-cooled PBS. The Annexin V-FITC/PI apoptosis detection kit (Solarbio, Beijing, China) was used for double staining. The cells were stained with Annexin V-FITC for 15 min and then propidium iodide (PI) in the dark for 5 min. Apoptosis analysis was performed with the flow cytometer (cytoFlexS, Beckman).

### 2.8 m^6^A quantitative analysis

TRIzol (Invitrogen) was utilized for total RNA extraction, and RNA quantity was examined using NanoDrop ND-1000. m^6^A RNA methylation quantification kit (ab185912, Abcam) was employed to test the m^6^A quality in total RNA, followed by the evaluation of absorbance at 450 nm.

### 2.9 m^6^A RNA immunoprecipitation quantitative polymerase chain reaction

MeRIP-qPCR was conducted as described in the previous literature (22) to quantify the level of m^6^A-modified LINC00958 in HeLa cells. Briefly, total RNA was extracted from CC cells using TRIzol. Purified RNA (5 µg) was digested by DNase I (M0303, NEB, Ipswich, MA, USA) and then incubated at 95°C for 25 s in RNA Fragmentation Reagents (AM8740, Ambion, Austin, Texas, USA), followed by ethanol precipitation and collection. Anti-m6A antibody (ab208577, Abcam) or anti-IgG (ab170190, Abcam) was incubated overnight with Protein A/G beads in IP buffer (150 mM NaCl, 0.1% NP-40, 10 mM Tris HCl, pH 7.4) at room temperature. RNA and prepared antibody-bead mixture were incubated in IP buffer at 4°C for 4 h. After three washes, the bound RNA was eluted from the beads with 0.5 mg/mL N6 methyladenosine (P3732, Berry&Associates) in IP buffer. The eluted RNA was extracted with Enol: Chloroform: Isoamylol (pH<5.0, P1025-500, Solarbio), and then cDNA was generated using All In One RT MasterMix (G490, ABM). The enrichment level of m6A was detected by reverse transcription quantitative polymerase chain reaction (RT-qPCR), with the control group level as the relative value.

### 2.10 RNA stability detection

HeLa cells were treated with 1 μg/mL actinomycin D. RNA was extracted for RT-qPCR at different times (0, 3, and 6 h).

### 2.11 RNA immunoprecipitation

The Magna RIP RNA-binding protein immunoprecipitation kit was obtained from Millipore (Billerica, MA, USA). More than 10^7^ SiHa or HeLa cells (23) were re-suspended in RIP lysis buffer with anti-c-MYC (ab32072, Abcam) or anti-IgG (ab172730, Abcam). Then, the cells in each group were added with magnetic bead and proteinase K. Finally, the precipitated RNA was isolated and purified for RT-qPCR.

### 2.12 Dual-luciferase assay

Two sets of luciferase reporter assay were performed. 1). To confirm whether LINC00958 regulated the transcriptional activity of c-MYC, we obtained c-MYC-responsive 4x-Ebox reporter vector from GenePharma and transfected it into SiHa or HeLa cells interfered with LINC00958. 2). To confirm whether BAG3 was the transcriptional target of c-MYC, we constructed the BAG3 promoter sequence fragments containing a c-MYC binding site (WT) or mutant site (MUT) and then inserted them into the pGL3 reporter vector respectively (Promega, Madison, WI, USA). The luciferase reporter plasmids were co-transfected with pcDNA3.1 c-MYC or pcDNA3.1 NC into SiHa or HeLa cells. The cells were lysed after 48 h. The luciferase activity was examined using the dual-luciferase reporter assay kit (Promega).

### 2.13 Chromatin immunoprecipitation

EZ ChIP kit (Millipore) was utilized for ChIP assay. Briefly, the treated SiHa or HeLa cells (1 × 10^7^) (24) were subjected to chromatin cross-linking, and DNA was cut into fragments by repeated ultrasound. Then, immunoprecipitation of DNA-protein complex was performed using the anti-c-MYC antibody (ab32072, Abcam) or IgG antibody (ab172730, Abcam). Afterward, the DNA of immunoprecipitated DNA-protein complex was extracted and purified using the DNA fragment purification kit (Intron Biotechnology, South Korea) for RT-qPCR, with GAPDH as NC.

### 2.14 Tumor xenograft assay

BALB/c nude mice (5-week-old) were obtained from Vital River Laboratory Animal Technology Co., Ltd (Beijing, China) [SYXK (Beijing) 2017-0033]. The mice were acclimated for 7 days and maintained under a 12-h dark/light cycle with sufficient food and water. HeLa cells were infected with LINC00958 shRNA lentivirus (Genechem) and the stably transfected cells were screened by 5 μg/mL puromycin. HeLa cells with stable LINC00958 knockdown or LINC00958 knockdown + METTL3 overexpression were collected and injected subcutaneously into mice on the right side near the forelimb (3 × 10^6^). From the 7^th^ day, the tumor growth was monitored weekly (Volume = Length × Width^2^/2). Four weeks after the injection, the mice were euthanized by intraperitoneal injection of 100 mg/kg pentobarbital sodium. The tumor was excised for subsequent analysis.

### 2.15 Immunohistochemistry

The tumor tissues were embedded in paraffin and sliced (5 μm). After dewaxing and rehydration, the antigen was retrieved with citrate buffer for 10 min. Then, the sections were subjected to incubation with 3% hydrogen dioxide for 15 min and normal goat serum (Solarbio) for 15 min. After that, the sections were incubated with METTL3 (ab195352, Abcam), c-MYC (ab32072, Abcam), and ki67 (ab15580, Abcam) at 4°C overnight, followed by incubation with the secondary antibody (ab205718, Abcam) at 37°C for 1 h. Finally, diaminobenzidine (Solarbio) and hematoxylin staining were conducted, followed by observation under a microscope (Olympus, Tokyo, Japan).

### 2.16 RT-qPCR

The gene expression in CC cells or tissues was quantitatively determined using RT-qPCR. TRIzol (Invitrogen) was used for RNA isolation. RNA concentration was measured using the Nano-Drop ND-1000 spectrophotometer. M-MLV (Takara, Kyoto, Japan) was employed for reverse transcription, and cDNA amplification was conducted using the SYBR Green Master Mix kit (Takara). **Table 1** exhibits the primers. The relative expression of genes was calculated using the 2^-ΔΔCt^ method (25), with GAPDH as the internal control.

**Table 1:** PCR primer sequence.

Name	Sequence (5’-3’)
LINC00958	F: CAGAGGTGAATGCAAGCTCAC
	R: ACAAAGGCAGAGCTTGAGCA
METTL3	F: ATGTCGGACACGTGGAGCTCTA
	R: GTCTAGTAGGTGGATCCCATC
c-MYC	F: AACAGGAACTATGACCTCGAC
	R: TTACGCACAAGAGTTCCGTAGC
BAG3	F: GGACCACAACAGCCGCACCAC
	R: CTACGGTGCTGCTGGGTTACC
GAPDH	F: ATGGTTTACATGTTCCAATATGA
	R: TTACTCCTTGGAGGCCATGTGG

### 2.17 Western blot

The protein was extracted using radio-immunoprecipitation assay buffer (Beyotime), separated by SDS-PAGE, and transferred onto PVDF membranes (Millipore). The membranes were blocked with skim milk for 2 h and incubated with the primary antibodies METTL3 (1:1000, ab195352, Abcam), c-MYC (1:1000, ab32072, Abcam), and GAPDH (1:2500, ab9485, Abcam) at 4°C overnight, followed by 3 times of tris-buffered saline-tween buffer (Solarbio) washing. Afterward, the membranes were incubated with the secondary antibody (1:2000, ab205718, Abcam) for 2 h. The gray value was analyzed using Image J (NIH, Bethesda, Maryland, USA).

### 2.18 Bioinformatics analysis

LINC00958 expression in CC, the relationships between c-MYC and LINC00958, and c-MYC and BAG3, and the correlation between c-MYC expression and the prognosis of CC patients were predicted through the GEPIA database (http://gepia.cancer-pku.cn/) (26). The prognosis of LINC00958 in CC and the expression of METTL3 and BAG3 in CC were predicted through the UALCAN database (http://ualcan.path.uab.edu/analysis.html) (27). The correlation between LINC00958 expression and the prognosis of CC patients was predicted through the Kaplan-Meier Plotter database (http://kmplot.com/analysis/index.php?p=service&cancer=liver_rnaseq) (28). The binding site of c-MYC and BAG3 promoter was predicted through the Jaspar website (http://jaspar.genereg.net/) (29). The downstream genes of c-MYC were predicted through the RNAInter database (http://www.rna-society.org/rnainter/) (30).

### 2.19 Statistical analysis

Data analysis and map plotting were performed using the SPSS 21.0 (IBM Corp., Armonk, NY, USA) and GraphPad Prism 8.0 (GraphPad Software Inc., San Diego, CA, USA). The data complied with the assumption of normality and homogeneity of variance. The measurement data are presented as mean ± standard deviation. The *t* test was used for comparisons between two groups. One-way or two-way analysis of variance (ANOVA) was employed for comparisons among multiple groups, followed by Tukey’s multiple comparison test or Sidak’s multiple comparison test. The enumeration counting data were presented as cases. Fisher exact test was utilized for comparisons between groups. Kaplan-Meier survival curve and Log-rank test were employed to determine the correlation between LINC00958 expression and the prognosis of CC patients. Pearson correlation analysis was utilized to determine the correlation between the factors. All *p*-values were two sided and a value of *p* < 0.05 was considered statistically significant.

## 3 Results

### 3.1 LINC00958 was upregulated in CC and correlated with the prognosis and clinicopathological features of CC patients

**Figure 1:**
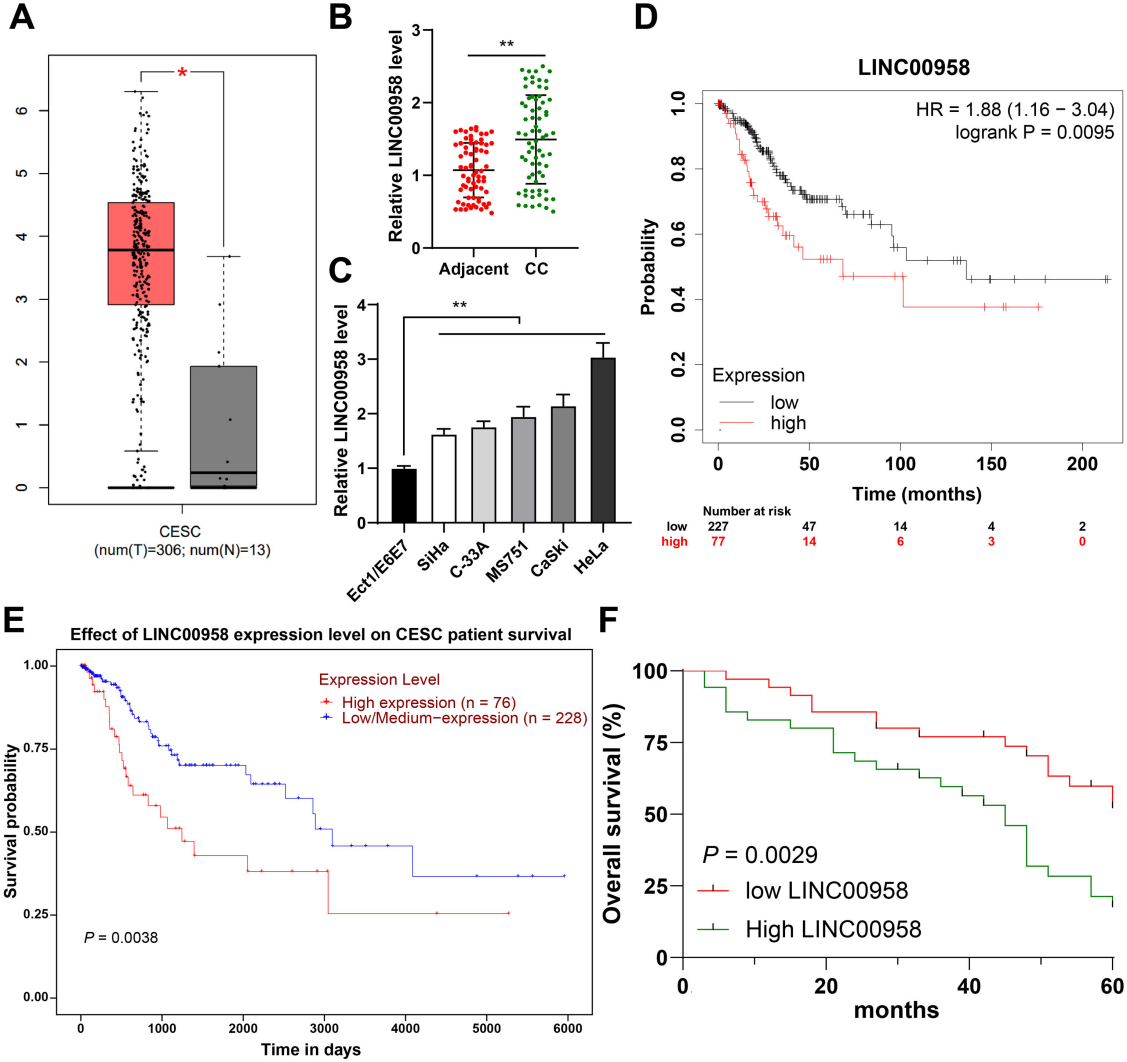
LINC00958 was highly expressed in CC and correlated with the prognosis and clinicopathological features of CC patients. **(A)** LINC00958 expression in CC was predicted through the GEPIA database. **(B)** LINC00958 expression in CC tissues and adjacent tissues was detected using RT-qPCR. **(C)** LINC00958 expression in cervical epithelial cells and CC cells was detected using RT-qPCR. **(D, E)** The correlation between LINC00958 expression and the prognosis of CC patients was predicted through the Kaplan-Meier Plotter and UALCAN databases. **(F)** The correlation between LINC00958 expression and the prognosis of CC patients was analyzed through the Kaplan-Meier survival curve. Clinical experiments N = 70, cell experiments N = 3. Data are presented as mean ± standard deviation. Data comparisons between two groups in **(B)** were performed using paired *t* test. Data in **(F)** were analyzed using Log-Rank test, and data in **(C)** were analyzed using one-way ANOVA, followed by Tukey’s multiple comparisons test, ^**^p < 0.01, *p < 0.05.

Considering that the role of LINC00958 in CC growth has not been fully elucidated, we predicted through the GEPIA database that LINC00958 exhibited an upregulation of expression in CC (**Figure 1A**). Our finding demonstrated that LINC00958 expression was elevated in CC tissues and cells (*p* < 0.01, **Figures 1B, C**). Then, we assigned CC patients to the LINC00958 high-expression group and LINC00958 low-expression group, with the median LINC00958 expression as the critical threshold (31). LINC00958 expression was correlated with tumor size, lymph node metastasis, and Federation of Gynecology and Obstetrics (FIGO) stage (*p* < 0.05, **Table 2**). Kaplan-Meier Plotter and UALCAN databases predicted that the survival of CC patients with high LINC00958 expression was notably shorter than that of CC patients with low LINC00958 expression (**Figures 1D, E**). Kaplan-Meier survival analysis of CC patients showed that higher LINC00958 expression indicated shorter overall survival (*p* < 0.01, **Figure 1F**). Altogether, LINC00958 was highly expressed in CC and correlated with the prognosis and clinicopathological features of CC patients.

**Table 2:** Correlation between LINC00958 expression and clinicopathological characteristics of cervical cancer patients.

Characteristic	Number	LINC00958		*p* value
		Low expression (N = 35)	High expression (N = 35)	
Age				
< 51	31	16	15	0.810
≥ 51	39	19	20	
Pathological type				
Squamous cell carcinoma	39	18	21	0.470
Adenocarcinoma	31	17	14	
Tumor size				
< 4 cm	42	26	16	0.015
≥ 4 cm	28	9	19	
Lymph node metastasis				
Negative	39	24	15	0.030
Positive	31	11	20	
Differentiation				
Well and moderately	35	15	20	0.232
Poor	35	20	15	
HPV infection				
Negative	37	20	17	0.473
Positive	33	15	18	
FIGO stage				
I	38	24	14	0.016
II	32	11	21	

Data comparison was performed using Fisher’s exact test.

FIGO, international federation of gynecology and obstetrics; HPV, human papillomavirus.

### 3.2 LINC00958 silencing suppressed CC cell proliferation and facilitated apoptosis

**Figure 2:**
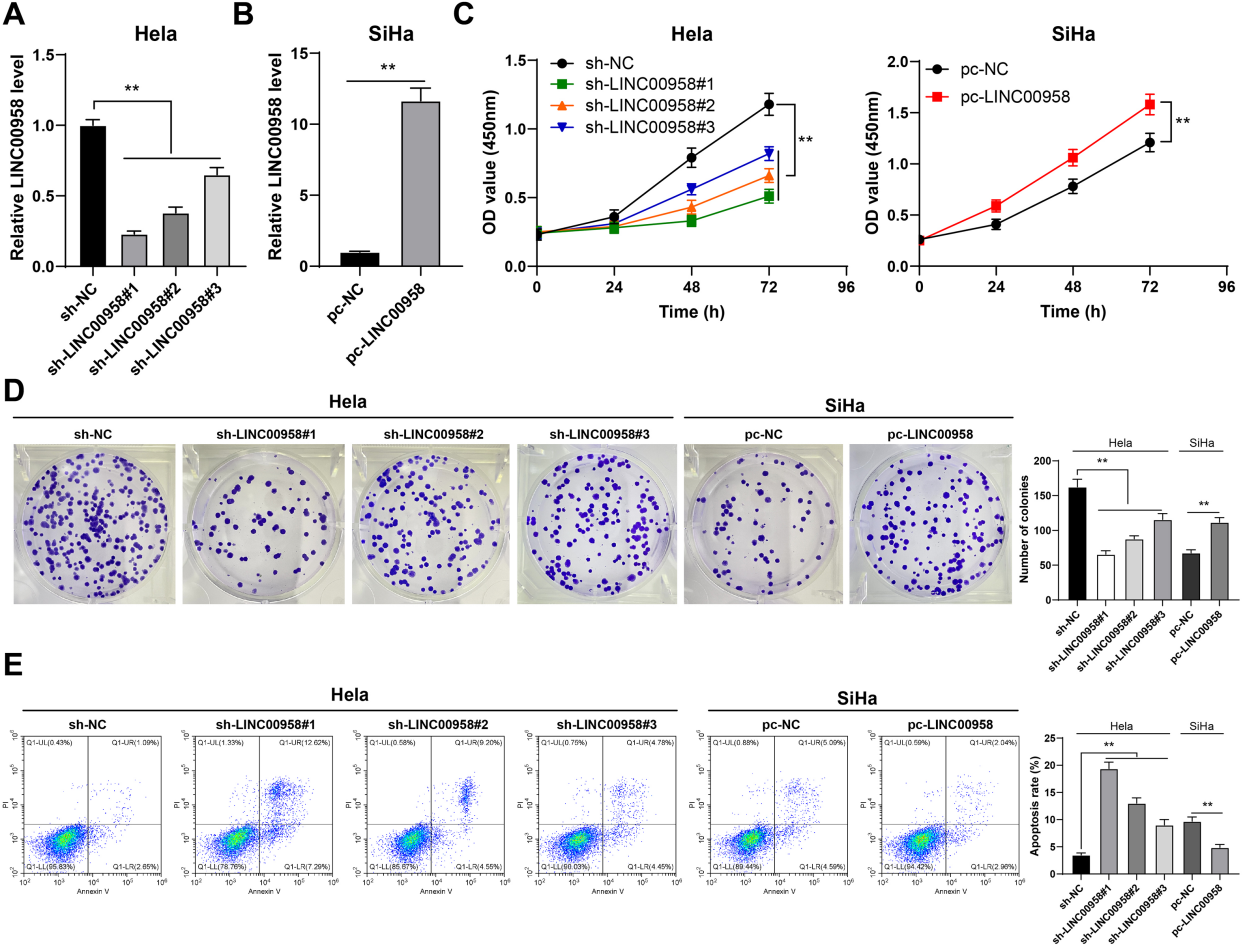
LINC00958 silencing suppressed CC cell proliferation and facilitated apoptosis. LINC00958 shRNA was transfected into HeLa cells and pcDNA3.1 LINC00958 was transfected into SiHa cells. **(A, B)** LINC00958 expression in cells was detected using RT-qPCR. **(C, D)** The proliferation of cells was measured using CCK-8 assay **(C)** and colony formation assay **(D)**. **(E)** The apoptosis of cells was measured using flow cytometry. Cell experiment N = 3 Data are presented as mean ± standard deviation. Data in **(B)** were analyzed using *t* test. Data comparisons between two groups in **(A, D, E)** were performed using *t* test. Data comparisons between multiple groups in **(A, D, E)** were performed using one-way ANOVA, and data comparisons between multiple groups in **(C)** were performed using two-way ANOVA, followed by Tukey’s multiple comparisons test or Sidak’s multiple comparisons test, ^**^p < 0.01. sh-NC: NC shRNA; sh-LINC00958: LINC00958 shRNA; pc-NC: pcDNA3.1 NC; pc-LINC00958: pcDNA3.1 LINC00958.

To explore the effect of LINC00958 on the proliferation and apoptosis of CC cells, we transfected sh-LINC00958 into HeLa cells with relatively high LINC00958 expression, and successfully downregulated LINC00958 expression in HeLa cells (*p* < 0.01, **Figure 2A**). pc-LINC00958 was transfected into SiHa cells with relatively low LINC00958 expression, and LINC00958 expression was successfully upregulated in SiHa cells (*p* < 0.01, **Figure 2B**). LINC00958 silencing reduced HeLa cell proliferation, while LINC00958 overexpression enhanced SiHa cell proliferation (*p* < 0.01, **Figures 2C, D**). LINC00958 silencing increased HeLa cell apoptosis, while LINC00958 overexpression decreased SiHa cell apoptosis (*p* < 0.01, **Figure 2E**). Briefly, LINC00958 silencing suppressed CC cell proliferation and facilitated apoptosis.

### 3.3 LINC00958 silencing repressed the growth of CC cells *in vivo*

**Figure 3:**
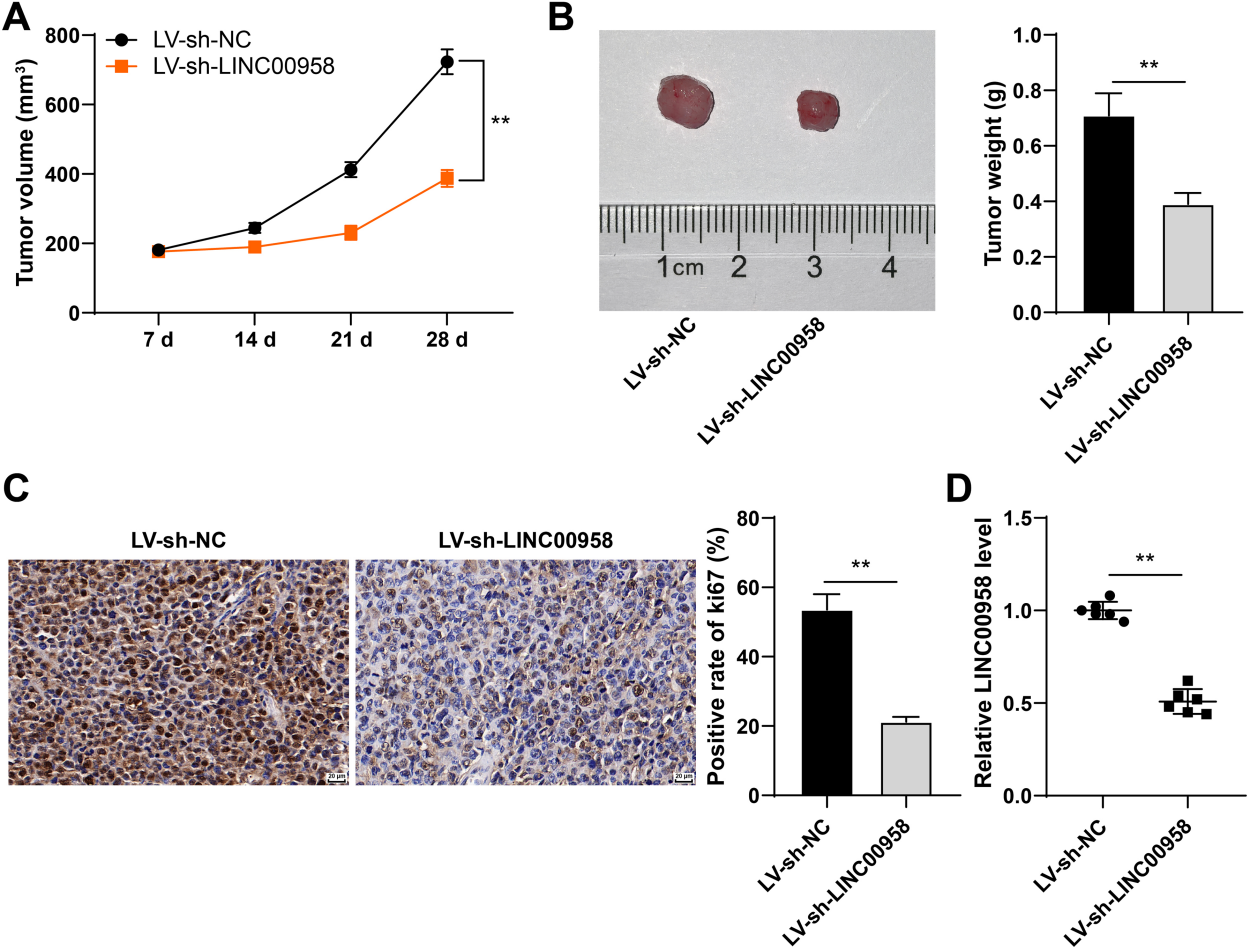
LINC00958 silencing repressed the growth of CC cells *in vivo.* HeLa cells with low expression of LINC00958 were used to establish the xenograft tumor model in nude mice. **(A)** Tumor volume. **(B)** On the 28^th^ day, the nude mice were euthanized, and the typical images and tumor weight were obtained. **(C)** The positive expression rate of ki67 was detected using immunohistochemistry. **(D)** LINC00958 expression in tumor tissues was detected using RT-qPCR. Animal experiments N = 6. Data are presented as mean ± standard deviation. Data comparisons between two groups in panels **(B–D)** were performed using *t* test. Data comparisons between multiple groups in **(A)** were performed using two-way ANOVA, followed by Sidak’s multiple comparisons test, ^**^p < 0.01. LV-sh-NC: lentivirus containing NC shRNA; LV-sh-LINC00958: lentivirus containing LINC00958 shRNA.

Subsequently, we established a nude mouse xenograft tumor model to evaluate the effect of LINC00958. LINC00958 silencing depressed tumor growth (*p* < 0.01, **Figure 3A**) and reduced tumor weight (*p* < 0.01, **Figure 3B**). Ki67 can reflect cell proliferation (28), so we employed immunohistochemistry to detect the positive rate of ki67 in tumors. LINC00958 silencing notably reduced the positive rate of ki67 (*p* < 0.01, **Figure 3C**). Compared with the LV-sh-NC group, the LV-sh-LINC00958 group showed reduced LINC00958 expression (*p* < 0.01, **Figure 3D**). These results indicated that LINC00958 silencing repressed the growth of CC cells *in vivo*.

### 3.4 METTL3-mediated m^6^A modification elevated LINC00958 expression in CC by promoting its RNA stability

**Figure 4:**
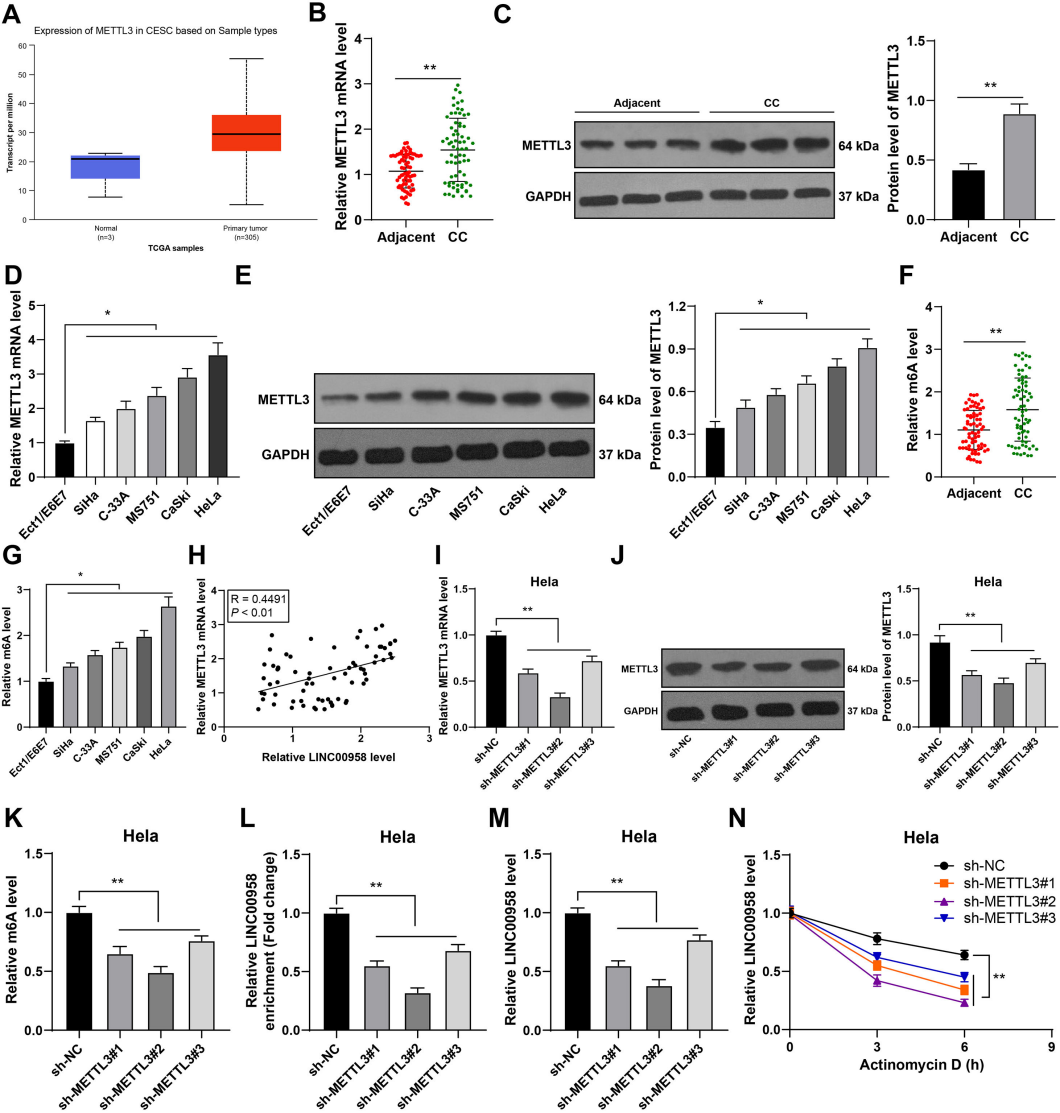
METTL3-mediated m^6^A modification upregulated LINC00958 expression in CC by promoting LINC00958 stability. **(A)** MTEEL3 expression in CC was predicted through the UALCAN database. **(B–E)** MTEEL3 expression in CC tissues and cells was detected using RT-qPCR and Western blot. **(F, G)** Quantitative analysis of m^6^A level in CC tissues and cells. **(H)** Pearson correlation analysis of MTEEL3 and LINC00958 in CC patients. Three METTL3 shRNAs were transfected into HeLa cells respectively, with as NC shRNA control. **(I, J)** MTEEL3 expression in CC cells was detected using RT-qPCR and Western blot. **(K)** Quantitative analysis of m^6^A level in cells. **(L)** m^6^A level of LINC00958 in cells was analyzed using MeRIP-qPCR. **(M)** LINC00958 expression in CC cells was detected using RT-qPCR. **(N)** The half-life of LINC00958 after treatment with actinomycin D for 0, 3, and 6 h was analyzed using RT-qPCR. Clinical experiments N = 70, cell experiments N = 3. Data are presented as mean ± standard deviation. Data comparisons between two groups in **(B, C, F)** were performed using paired *t* test. Data comparisons between multiple groups in **(D, E, G, I–M)** were performed using one-way ANOVA, and data comparisons between multiple groups in **(N)** were performed using two-way ANOVA, followed by Tukey’s multiple comparisons test, ^*^p < 0.05, ^**^p < 0.01. sh-NC: NC shRNA; sh-METTL3: METTL3 shRNA.

Then, we focused on the downstream mechanism of LINC00958 in CC growth. METTL3-mediated m^6^A modification regulates LINC00958 expression in hepatocellular carcinoma (19). UALCAN database predicted the upregulation of METTL3 expression in CC (**Figure 4A**), and our results also confirmed the elevation of METTL3 expression in CC tissues and cells (*p* < 0.05, **Figures 4B–E**). m^6^A quantitative analysis showed that the m^6^A level in CC tissues and cells was consistent with the trend of METTL3 expression (*p* < 0.05, **Figures 4F, G**). METTL3 expression was positively correlated with LINC00958 expression in CC tissues (*p* < 0.01, **Figure 4H**). We speculated that the upregulation of LINC00958 expression in CC was related to METTL3-mediated m6A modification. We successfully downregulated METTL3 expression in HeLa cells by transfecting sh-METTL3 (*p* < 0.01, **Figures 4I, J**), and found that the m^6^A level was also decreased (*p* < 0.01, **Figure 4K**). Additionally, METTL3 silencing decreased m^6^A level in LINC00958 (*p* < 0.01, **Figure 4L**) and reduced LINC00958 expression (*p* < 0.01, **Figure 4M**). Moreover, we treated HeLa cells with actinomycin D to block transcription, and found that METTL3 silencing notably shortened the half-life of LINC00958 (*p* < 0.01, **Figure 4N**). Briefly, METTL3-mediated m^6^A modification elevated LINC00958 expression in CC by promoting its RNA stability.

### 3.5 METTL3 overexpression offset the impact of LINC00958 silencing on CC cells

**Figure 5:**
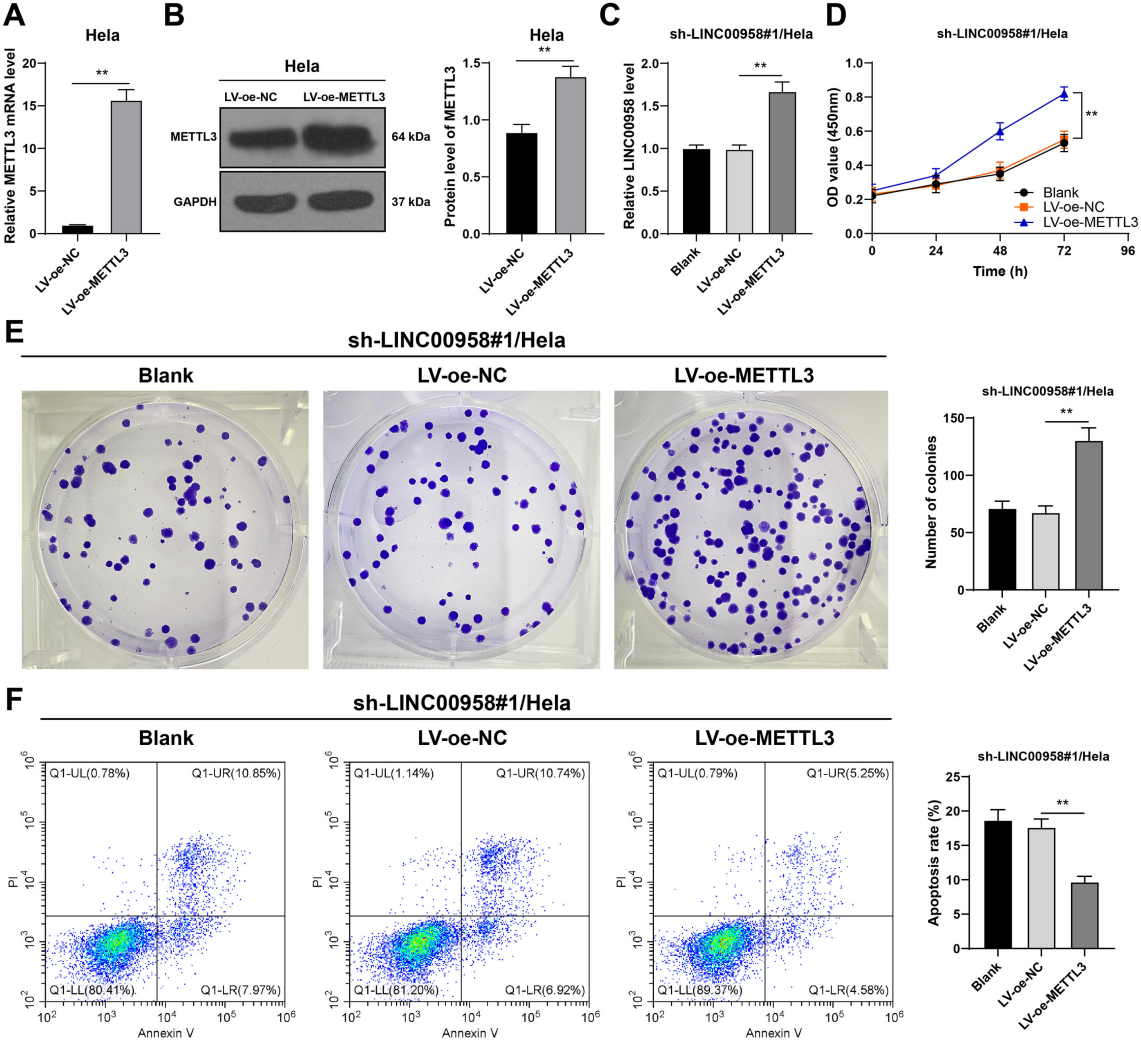
METTL3 overexpression attenuated the inhibitory effect of LINC00958 silencing on CC growth. HeLa cells were infected with LV-oe-METTL3. **(A, B)** MTEEL3 expression in CC cells was detected using RT-qPCR and Western blot. Then, LV-oe-METTL3-treated HeLa cells were infected with sh-LINC00958#1. **(C)** LINC00958 expression in cells was detected using RT-qPCR. **(D, E)** The proliferation of cells was measured using CCK-8 assay **(D)** and colony formation assay **(E). (F)** The apoptosis of cells was measured using flow cytometry. Cell experiment N = 3. Data are presented as mean ± standard deviation. Data comparisons between two groups in **(A, B)** were performed using *t* test. Data comparisons between multiple groups in **(C, E, F)** were analyzed using one-way ANOVA, and data comparisons between multiple groups in **(D)** were performed using two-way ANOVA, followed by Tukey’s multiple comparisons test, ^**^p < 0.01. LV-sh-LINC00958: LINC00958 shRNA; LV-oe-METTL3: lentiviral overexpression vector of METTL3; LV-oe-NC: lentiviral overexpression vector of NC.

To verify the role of METTL3/LINC00958 axis in CC, we designed a functional rescue experiment. HeLa cells were infected with LV-oe-METTL3 to upregulate METTL3 expression (*p* < 0.01, **Figures 5A, B**), and then the HeLa cells were further treated with sh-LINC00958#1. Compared with LINC00958 silencing alone, the combined treatment of METTL3 overexpression and LINC00958 silencing increased LINC00958 expression in cells (*p* < 0.01, **Figure 5C**), enhanced cell proliferation (*p* < 0.01, **Figures 5D, E**), and decreased apoptosis (*p* < 0.01, **Figure 5F**). Briefly, METTL3 overexpression offset the impact of LINC00958 silencing on CC growth.

### 3.6 LINC00958 activated c-MYC transcriptional activity

**Figure 6:**
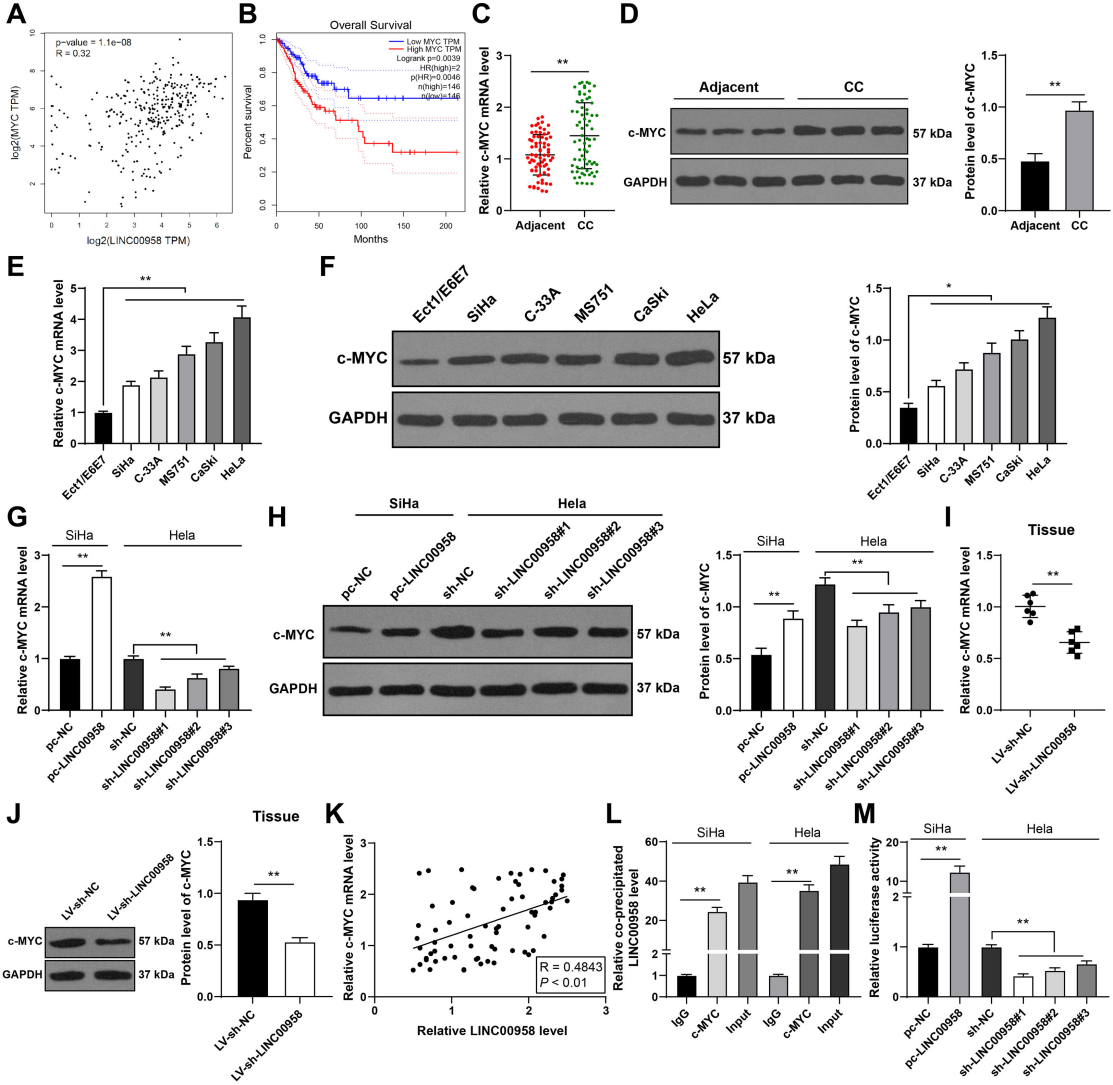
LINC00958 activated the transcriptional activity of c-MYC. **(A, B)** The correlation between c-MYC and LINC00958 and the correlation between c-MYC level and prognosis of CC patients were predicted through the GEPIA database. **(C–J)** c-MYC expression in CC tissues and cells was detected using RT-qPCR and Western blot. **(K)** Pearson correlation analysis of c-MYC and LINC00958 in CC patients. **(L)** The binding of LINC00958 and c-MYC was analyzed using RIP assay. **(M)** The luciferase activity of c-MYC responsive construct was detected using dual-luciferase reporter assay. Clinical experiments N = 70, cell experiments N = 3, animal experiments N = 6. Data are presented as mean ± standard deviation. Data comparisons between two groups in **(C, D)** were performed using paired *t* test, and data comparisons between two groups in panels **(G–J, M)** were performed using *t* test. Data comparisons between multiple groups in **(E–H, L, M)** were performed using one-way ANOVA, followed by Tukey’s multiple comparisons test, ^*^p < 0.05, ^**^p < 0.01. LV-sh-NC: lentivirus containing NC shRNA; LV-sh-LINC00958: lentivirus containing LINC00958 shRNA; sh-NC: NC shRNA; sh-LINC00958: LINC00958 shRNA; pc-NC: pcDNA3.1 NC; pc-LINC00958: pcDNA3.1 LINC00958.

Thereafter, the downstream mechanism of LINC00958 was investigated. LINC00958 can activate c-MYC transcription in head and neck squamous cell carcinoma (HNSCC) (32). GEPIA database predicted that LINC00958 expression was positively correlated with c-MYC expression in CC (**Figure 6A**), and patients with higher c-MYC expression had shorter survival (**Figure 6B**). Hence, we speculated whether LINC00958 played a role in CC by activating the transcriptional activity of c-MYC. Our results demonstrated that c-MYC expression was elevated in CC tissues and cells (*p* < 0.01, **Figures 6C–F**), and LINC00958 overexpression increased c-MYC expression, while LINC00958 silencing decreased c-MYC expression (*p* < 0.01, **Figures 6G–J**). c-MYC expression was positively correlated with LINC00958 expression in CC tissues (*p* < 0.01, **Figure 6K**). Moreover, RIP results showed that compared with IgG, LINC00958 was highly enriched in c-MYC complex (*p* < 0.01, **Figure 6L**). LINC00958 overexpression induced the luciferase activity of c-MYC responsive construct, while LINC00958 silencing showed an opposite trend in HeLa cells (*p* < 0.01, **Figure 6M**). Altogether, LINC00958 activated the transcriptional activity of c-MYC in CC.

### 3.7 c-MYC bound to the BAG3 promoter to promote its transcription

**Figure 7:**
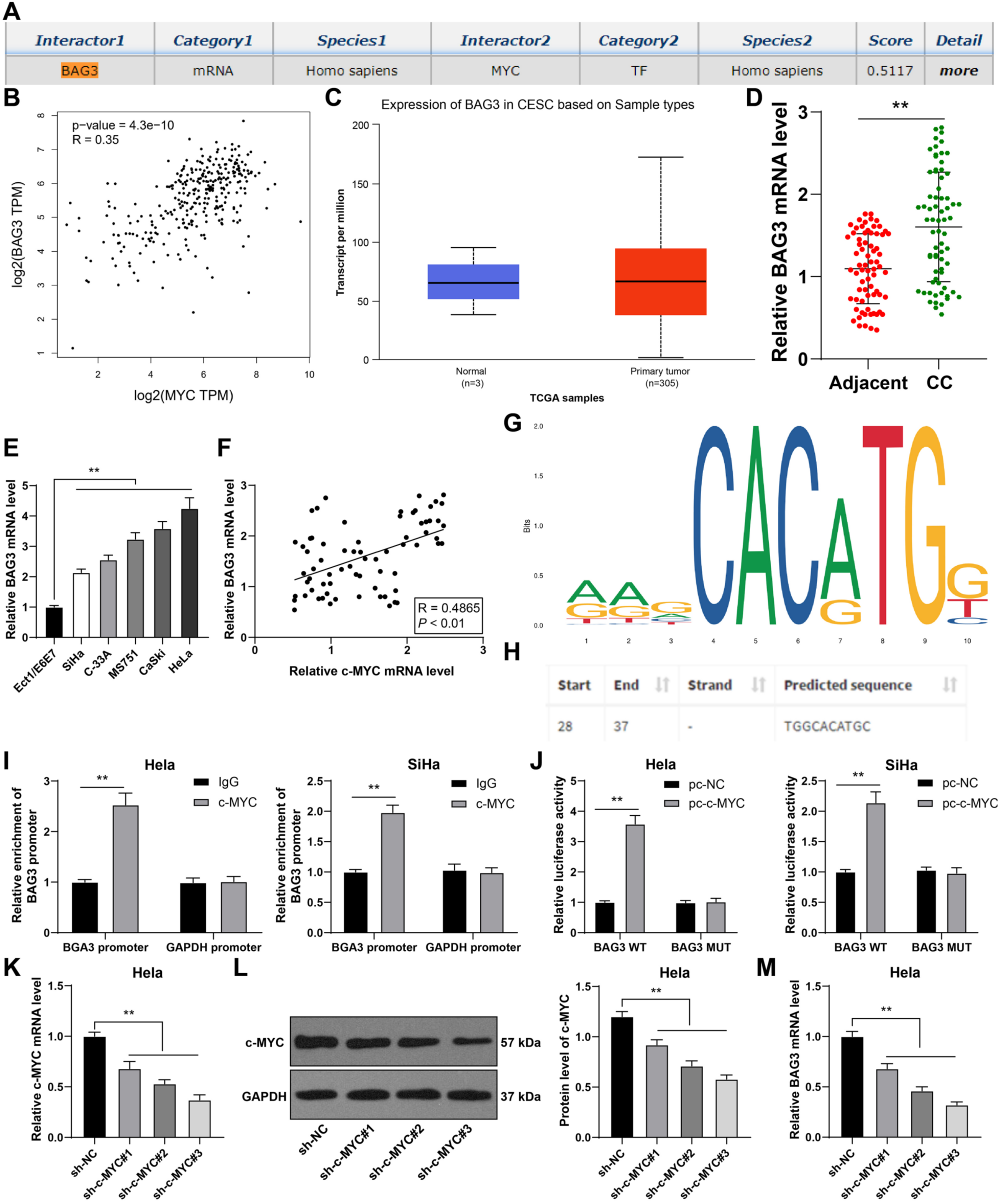
c-MYC bound to BAG3 promoter to promote its transcription. **(A)** The binding relationship between c-MYC and BAG3 was predicted through the RNAInter database. **(B)** The correlation between BAG3 and c-MYC was predicted through the GEPIA database. **(C)** BAG3 expression in CC was analyzed through the UALCAN database. **(D, E)** BAG3 mRNA expression in CC tissues and cells was detected using RT-qPCR. **(F)** Pearson correlation analysis of c-MYC and BAG3 in CC patients. **(G, H)** The binding site of c-MYC to BAG3 promoter was predicted through the Jaspar website. **(I, J)** The binding relationship between c-MYC and BAG3 was verified using ChIP and dual-luciferase reporter assay. Three c-MYC shRNAs were transfected into HeLa cells respectively, with NC shRNA as control. **(K, L)** c-MYC expression was detected using RT-qPCR and Western blot. **(M)** BAG3 mRNA expression in cells was detected using RT-qPCR. Clinical experiments N = 70, cell experiments N = 3. Data are presented as mean ± standard deviation. Data comparisons between two groups in **(D)** were performed using *t* test. Data comparisons between multiple groups in **(E, K–M)** were performed using one-way ANOVA, followed by Tukey’s multiple comparisons test, and data comparisons between multiple groups in **(I, J)** were performed using two-way ANOVA, followed by Sidak’s multiple comparisons test, ^**^p < 0.01. pc-NC: pcDNA3.1 NC; pc-c-MYC: pcDNA3.1 c-MYC; sh-NC: NC shRNA; sh-c-MYC: c-MYC shRNA.

As a transcription factor, c-MYC can activate mRNA (33). The downstream genes of c-MYC were predicted through the RNAInter database, among which the apoptosis-related factor BAG3 is highly expressed in CC (**Figure 7A**) and concerned with CC cell proliferation and apoptosis (32–34). GEPIA database predicted that c-MYC was positively correlated with BAG3 in CC (**Figure 7B**). UALCAN database predicted an elevation of BAG3 expression in CC (**Figure 7C**). We speculated that BAG3 was a downstream mechanism of cMYC. Our results also revealed that BAG3 expression was elevated in CC tissues and cells (*p* < 0.01, **Figures 7D, E**), and c-MYC expression was positively correlated with BAG3 expression in CC tissues (*p* < 0.01, **Figure 7F**). Additionally, ChIP and dual-luciferase assay were designed according to the binding site of c-MYC and BAG3 promoter obtained from the Jaspar website (*p* < 0.01, **Figures 7G, H**). The results confirmed that c-MYC could bind to BAG3 promoter (*p* < 0.01, **Figures 7I, J**). Then, c-MYC was silenced in HeLa cells to further explore the effect of c-MYC on BAG3 transcription. RT-qPCR and Western blot results confirmed that the three c-MYC shRNAs had high intervention efficiency (*p* < 0.01, **Figures 7K, L**). BAG3 mRNA expression was decreased after c-MYC silencing (*p* < 0.01, **Figure 7M**). Briefly, c-MYC bound to the BAG3 promoter to promote its transcription.

### 3.8 BAG3 overexpression offset the impact of LINC00958 silencing on CC growth

**Figure 8:**
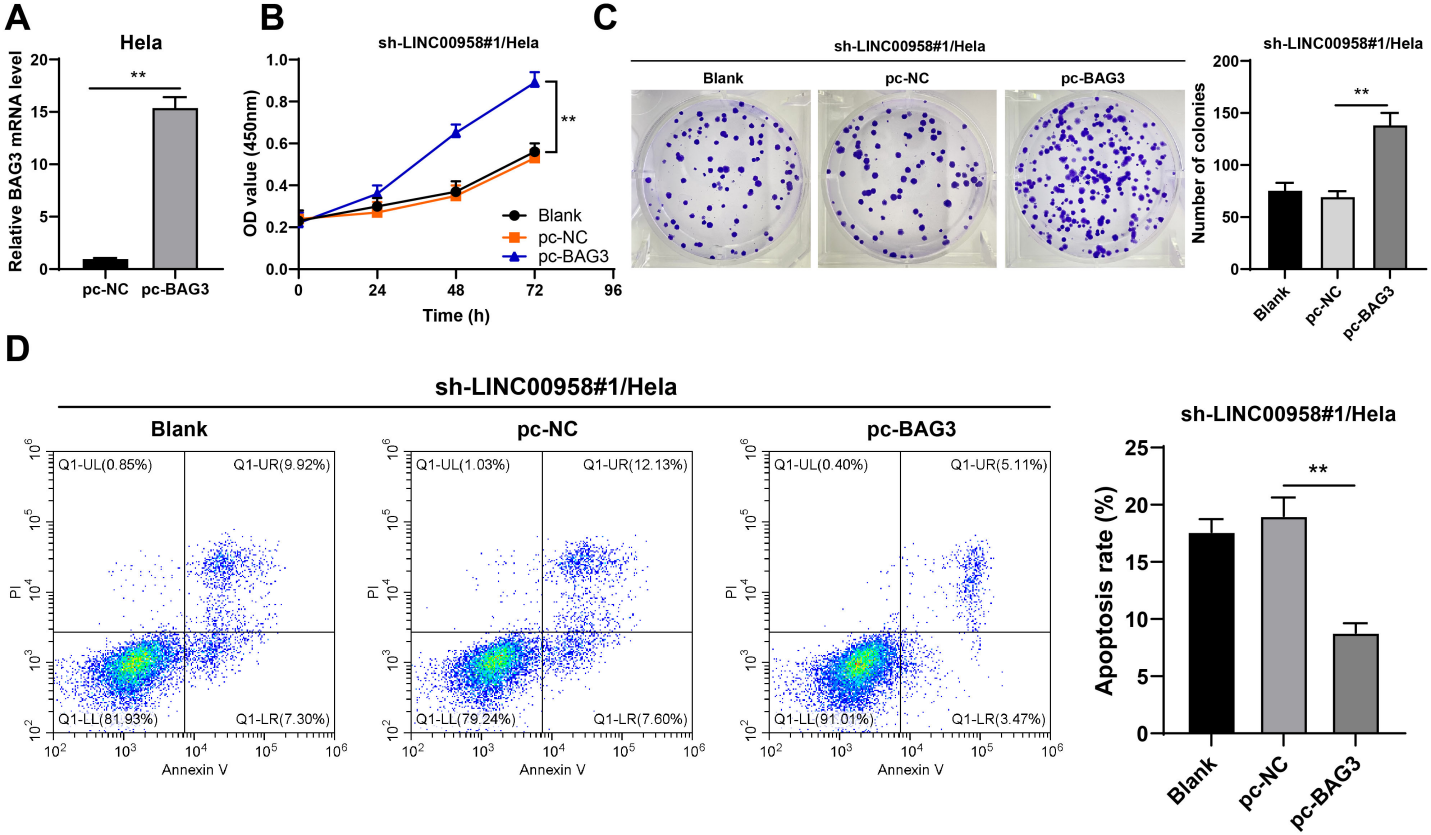
BAG3 overexpression reversed the inhibitory effect of LINC00958 silencing on CC growth. pcDNA3.1 BAG3 was transfected into HeLa cells, with pcDNA3.1 NC as control. **(A)** BAG3 mRNA expression in HeLa cells was detected using RT-qPCR. Then, pcDNA3.1 BAG3-treated HeLa cells were transfected with sh-LINC00958#1. **(B, C)** The proliferation of cells was measured using CCK-8 assay **(B)** and colony formation assay **(C). (D)** The apoptosis of cells was measured using flow cytometry. Cell experiments N = 3. Data are presented as mean ± standard deviation. Data comparisons between two groups in **(A)** were performed using *t* test. Data comparisons between multiple groups in **(C, D)** were performed using one-way ANOVA, and data comparisons between multiple groups in **(B)** were performed using two-way ANOVA, followed by Tukey’s multiple comparisons test, ^**^p < 0.01. sh-NC: NC shRNA; sh-LINC00958: LINC00958 shRNA; pc-NC: pcDNA3.1 NC; pc-BAG3: pcDNA3.1 BAG3.

The effect of LINC00958/BAG3 axis on CC cell growth was verified. pc-BAG3 was transfected into cells to upregulate BGA3 mRNA expression (*p* < 0.01, **Figure 8A**), and then the cells were further treated with sh-LINC00958#1. Compared with LINC00958 silencing alone, the combined treatment of LINC00958 silencing and BAG3 overexpression enhanced cell proliferation (*p* < 0.01, **Figures 8B, C**) and reduced apoptosis (*p* < 0.01, **Figure 8D**). Briefly, BAG3 overexpression offset the impact of LINC00958 silencing on CC growth.

### 3.9 METTL3 accelerated CC growth *in vivo* via the LINC00958/c-MYC/BAG3 axis

**Figure 9:**
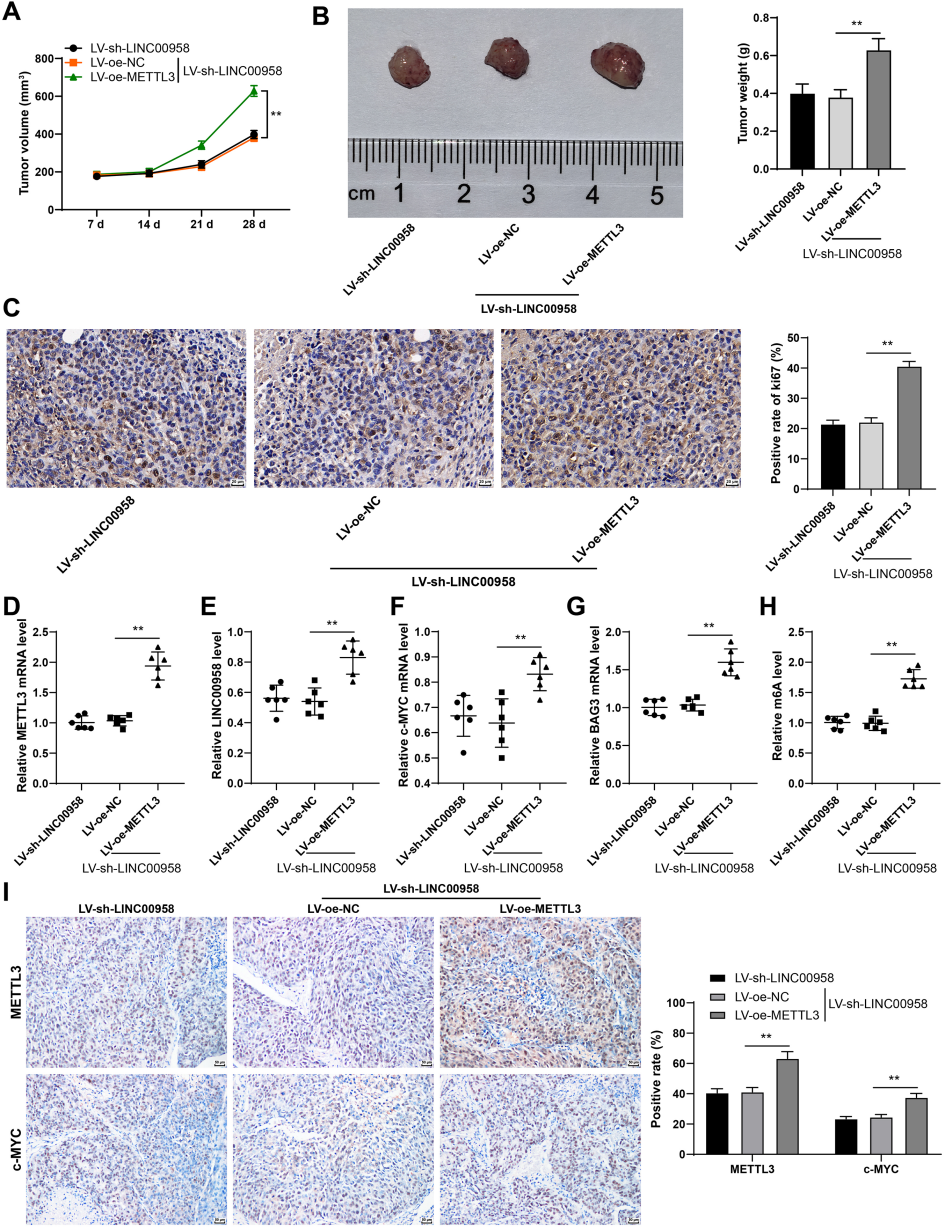
METTL3 promoted the growth of CC cells *in vivo* via the LINC00958/c-MYC/BAG3 axis. HeLa cells with stable METTL3 overexpression and LINC00958 low expression were used to establish the xenograft tumor model in nude mice. **(A)** Tumor volume. **(B)** On the 28^th^ day, the nude mice were euthanized, and the typical images and tumor weight were obtained. **(C)** The positive expression rate of ki67 was detected using immunohistochemistry. **(D–G)** METTL3, LINC00958, c-MYC, and BAG3 expressions were detected using RT-qPCR. **(H)** Quantitative analysis of m^6^A level. **(I)** The levels of METTL3 and c-MYC were analyzed using immunohistochemistry. Animal experiments N = 6. Data are presented as mean ± standard deviation. Data comparisons between multiple groups in **(B–H)** were performed using one-way ANOVA, and data comparisons between multiple groups in **(A, I)** were performed using two-way ANOVA, followed by Tuke’s multiple comparisons test, ^**^p < 0.01. LV-sh-NC: lentivirus containing NC shRNA; LV-sh-LINC00958: lentivirus containing LINC00958 shRNA; LV-oe-METTL3: lentiviral overexpression vector of METTL3; LV-oe-NC: lentiviral overexpression vector of NC.

Finally, we verified the role of METTL3 in CC growth and its regulatory effect on the c-MYC/BAG3 axis *in vivo*. METTL3 overexpression reduced the inhibitory effect of LINC00958 silencing on tumor growth, manifested as notably increased tumor volume and weight (*p* < 0.01, **Figures 9A, B**) and elevated ki67-positive rate (*p* < 0.01, **Figure 9C**). Compared with the sh-LINC00958#1 + oe-NC group, the sh-LINC00958#1 + oe-METTL3 group showed increased expressions of METTL3, LINC00958, c-MYC, and BAG3 in tumor tissues (*p* < 0.01, **Figures 9D–G**), and elevated m^6^A level (*p* < 0.01, **Figure 9H**). Immunohistochemical results also exhibited the same trend (*p* < 0.01, **Figure 9I**). Briefly, METTL3 accelerated the growth of CC cells *in vivo* via the LINC00958/c-MYC/BAG3 axis.

## 4 Discussion

**Figure 10:**
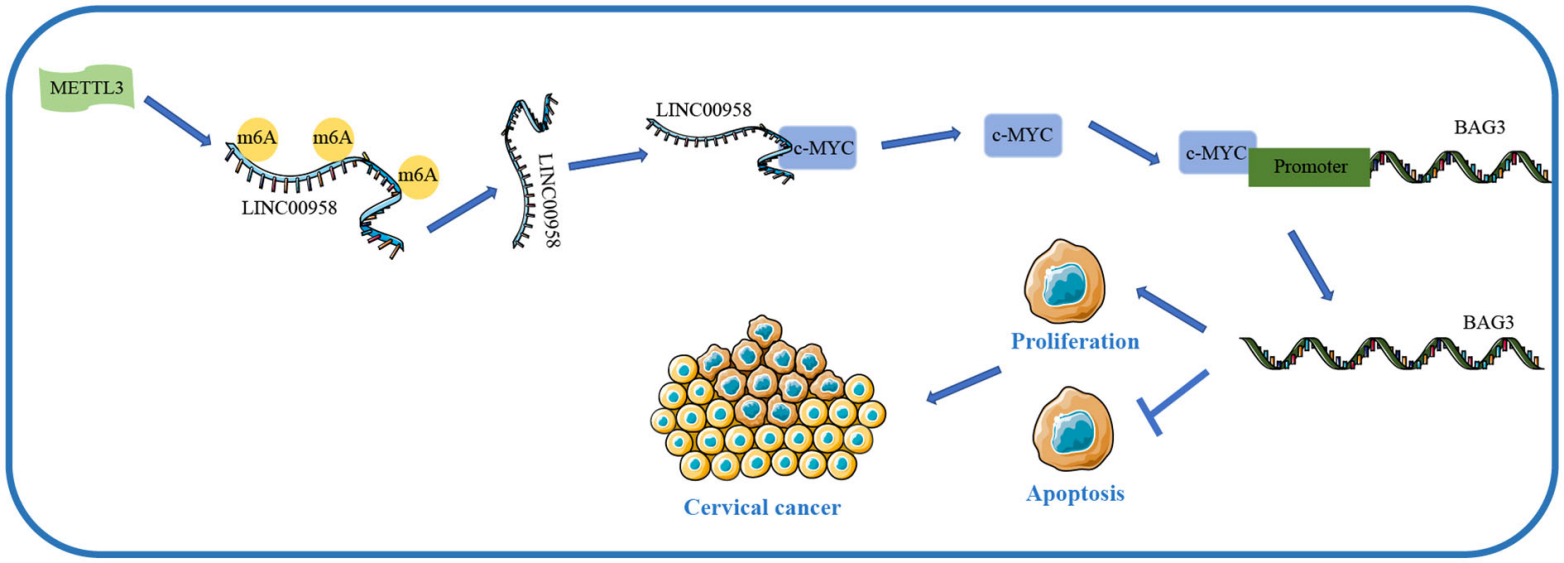
Mechanism of LINC00958 in CC growth. METTL3-mediated m^6^A modification can increase the stability of LINC00958 and upregulate the expression of LINC00958 in CC. LINC00958 can activate the transcription activity of c-MYC, and c-MYC can bind to BAG3 promoter and upregulate its transcription level, thereby promoting the proliferation of CC cells, inhibiting apoptosis, and ultimately promoting the growth of CC.

Although strenuous efforts have been invested for the treatment of CC, the clinical outcomes of CC patients are still dismal (35). As lncRNAs are commonly dysregulated in cervical malignancies, elucidating the role of lncRNA in CC growth is conducive to the development of effective treatment strategies (36). Emerging evidence has suggested that METTL3-mediated m^6^A modification affects RNA metabolism and participates in the pathogenesis of cancers (37). This study elucidated that METTL3-mediated m^6^A modification elevates LINC00958 expression, and LINC00958 enhances CC proliferation but represses apoptosis via the c-MYC/BAG3 axis (**Figure 10**).

LINC00958 has been reported as an oncogene in CC (11). LINC00958 also affects the radiosensitivity of CC cells, and higher LINC00958 results in poorer outcomes in CC patients (38). However, the exact regulatory mechanism of LINC00958 in CC growth remained largely unknown. In the current study, our findings consistently exhibited that LINC00958 was highly expressed in CC patients, and higher expression of LINC00958 was correlated with shorter overall survival. The upregulation of LINC00958 facilitates CC cell proliferation and metastasis by sponging miR-625-5p (11). We also revealed that LINC00958 silencing notably reduced proliferation and enhanced apoptosis of HeLa cells, while LINC00958 overexpression led to an opposite trend in SiHa cells. *In vivo* results confirmed that LINC00958 silencing repressed tumor growth and reduced ki67-positive rate in nude mice. Overall, silencing LINC00958 depressed CC cell proliferation and facilitated apoptosis *in vitro*, and also repressed tumor growth *in vivo*.

Subsequently, we sought to determine the upstream mechanism of LINC00958 in CC growth. m^6^A is acknowledged as the most frequent modification in lncRNAs (39). m^6^A modification mediated by methyltransferase METTL3 promotes tumorigenesis and Warburg effect in CC (35). Importantly, METTL3-mediated m^6^A modification results in LINC00958 upregulation by stabilizing RNA transcript (19). Accordingly, we speculated that the upregulation of LINC00958 in CC was related to METTL3-mediated m^6^A modification. Our results exhibited that METTL3 expression and m6A level were increased in CC tissues and cells, while silence of METTL3 in Hela cells reduced m^6^A level in LINC00958 and diminished LINC00958 expression, accompanied by shortened half-life of LINC00958, suggesting that METTL3-mediated m^6^A modification elevated LINC00958 expression by enhancing its RNA stability. METTL3 knockdown can repress CC proliferation through post-transcriptional regulation of RAB2B (40). Similarly, our functional rescue experimental findings indicated that METTL3 overexpression attenuated the inhibitory effect of LINC00958 silencing on the growth of CC cells.

Thereafter, we determined the downstream mechanism of LINC00958 in CC. c-MYC is the most characterized proto-oncogene abnormally activated in human cancers through chromosome translocation, gene amplification, and upstream carcinogenic signals (41, 42). LINC00958 is a direct target of c-MYC, which can strengthen c-MYC transcriptional activity and regulate the radiotherapy tolerance of HNSCC cells (43). Our results confirmed that c-MYC expression was elevated in CC, and LINC00958 activated the transcriptional activity of c-MYC.

The transformation activity of c-MYC is commonly believed to depend on its ability to regulate numerous genes involving in diverse cellular functions (44). BAG3 is a co-chaperone protein highly expressed in numerous cancer cells, skeletal muscle, and cardiomyocytes (45). BAG3 can modulate the levels, localization, or activity of its partner proteins, thereby regulating major cell functions, including apoptosis, autophagy, and cytoskeleton organization (46). BAG3 is implicated in the EMT process of CC, including cell growth, invasion, and migration (32). Overexpression of BAG3 induces CC cell survival and proliferation through the alteration of gene transcription (47). Our results demonstrated that BAG3 expression was elevated in CC, showing a positive correlation with c-MYC. The combined treatment of BAG3 overexpression and LINC00958 silencing enhanced CC cell proliferation and suppressed apoptosis, indicating that BAG3 overexpression attenuated the regulatory effect of LINC00958 silencing on CC cell growth. Consistently, BAG3 silencing reduces the expressions of EMT biomarkers and suppresses tumor growth in CC mice to retard the aggressive progression (32). Finally, we verified the role of METTL3 in the promotion of CC growth by LINC00958 and its regulatory effect on the c-MYC/BAG3 axis *in vivo*. Our results exhibited that METTL3 overexpression enhanced tumor growth, and elevated the levels of LINC00958, c-MYC, BAG3, and m^6^A. Briefly, METTL3 promoted CC growth *in vivo* via the LINC00958/c-MYC/BAG3 axis.

## 5 Conclusion

METTL3-mediated m^6^A modification elevated LINC00958 expression by enhancing its RNA stability, and LINC00958 activated the transcriptional activity of c-MYC. c-MYC bound to BAG3 promoter to enhance its transcription, thereby facilitating CC cell proliferation and repressing apoptosis.

However, several limitations warrant discussion. First of all, we did not determine the specific m6A site on LINC00958, and also failed to clarify the reading protein that mediates the expression of LINC00958. Secondly, this study merely explored the effect of BAG3 mRNA expression on CC, and the regulation of BAG3 protein level remains to be studied. Thirdly, whether METTL3 and c-MYC can co-regulate LINC00958 in CC is unknown. Last but not least, LINC00958 is predicted to be located in the cytoplasm, and its competing endogenous RNA mechanism needs to be explored. In the future, we will explore the upstream mechanism of LINC00958 and determine the specific m6A modification site and m6A reading protein of LINC00958. Moreover, we will verify the effect of BAG3 protein level on CC and explored other potential mechanisms of LINC00958 in CC to provide new theoretical knowledge for the treatment of CC.

## Data Availability

The datasets presented in this study can be found in online repositories. The names of the repository/repositories and accession number(s) can be found in the article/supplementary material.
